# Outpatient compulsory observation and treatment by a psychiatrist: Russian perspective

**DOI:** 10.1192/j.eurpsy.2025.1519

**Published:** 2025-08-26

**Authors:** V. V. Motov

**Affiliations:** 1 Psychiatry, Social House “Stupino” (The Institution for Chronic Mental Patients with Disability) of The Department of Labor and Social Protection of the Population of the City of Moscow, Stupino, Russian Federation

## Abstract

**Introduction:**

Outpatient compulsory observation and treatment by a psychiatrist (OCOTP) is, according to Russian Law, a coercive measure of medical nature. It was put into effect in 1997 and may be assigned by the court to persons suffering from mental disorders who have committed criminal offenses. Forensic psychiatric examination is a mandatory condition for the court to make a decision.

**Objectives:**

To provide an overview of (1) the purposes of OCOTP, (2) the differences between OCOTP and outpatient mandatory psychiatric treatment, (3) the basis for judicial decisions to order and terminate OCOTP, the duration of OCOTP, and its effectiveness.

**Methods:**

An analysis of scientific publications and professional literature on the topic and my own practical experience as a forensic psychiatrist who recommended such a measure to the court, and the treating psychiatrist who carried it out in a mental institution.

**Results:**

OCOTP may be imposed by the court (1) in relation to defendants found NGRI, who committed socially dangerous acts primarily in a state of “temporary mental disorder”, (2) following completion of compulsory inpatient treatment of an insane person, (3) as a preventive measure in conjunction with punishment when the offender is found guilty but has a mental disorder that can be treated on an outpatient basis. OCOTP is ordered for an indeterminate period, and the court considers its extension or termination annually.

**Conclusions:**

OCOTP is an evolving concept and the topic of discussion in professional literature.

**Disclosure of Interest:**

None Declared

